# Institutional Needs

**Published:** 1913-04-05

**Authors:** 


### Institutional Needs.

### INSTANTANEOUS ICES.

Messrs. Hanneford, Limited, of 91 and 93 Queen Victoria Street, London, E.C., have put on the market a machine which they guarantee to freeze ices in less than thirty seconds. It is known as the "Kuik" Freezer, and is designed so that a child may work it and that it will deliver a continuous supply of ice-cream ready for use. A full charge requires only 4 lb. of ice, while between one and two quarts is obtainable for about half this quantity. Institutional staffs understand fête organisation, on the big and the small scale, too well not to appreciate a machine which will make what are actually instantaneous ices.

### THE "VICTORIA" VEGETABLE-PEELER.

The installation of labour-saving machinery, where such is really a save, is typical of modern municipal administration, and a first-rate medical superintendent will be found as proud of his institutional kitchen as of his wards or his theatre. The peeling of vegetables by hand has long been a laborious and wasteful process, and a very good device in place of hand-labour is the "Victoria" Vegetable-Paring Machine, which a large number of institutions adopt for the peeling of potatoes, carrots, turnips, and similar root-vegetables. In referring to this machine last year in an article on up-to-date kitchen specialties, it was remarked: "In this machine the perforated metal sheet of the old nutmeg-grater type is replaced by a lining of granular carborundum," which not only adds to the life of the apparatus, but prevents waste or mess in the kitchen. Success always brings imitators, and machines have been put upon the market purporting to do the same work in the same manner; but the Imperial Machine Company, of 11 Crown Parade, Cricklewood, who are the manufacturers of the "Victoria" Vegetable-Paring Machines, have protected their apparatus by means of five British patents. One of these consists of a basic patent covering the process of incorporating carborundum with metal while in a molten or semi-molten condition, so that in competing machines the carborundum is only fastened on to the metal with enamels or cements, and after a time comes away in patches and becomes useless, whereas in the "Victoria" Machine this lasts for several years without renewal.

### THE "EUREKA" CREPE VELPEAU BANDAGE.

This bandage has a special interest from the fact that it contains no rubber, while giving the cool, elastic, and porous virtues associated so uncritically with rubber substitutes. Mr. Vincent Wood, surgical-appliance maker, Victoria House, Albion Place, Blackfriars Bridge, S.E., is the person from whom it can be obtained, and we have noticed with approval before how its permanent elasticity, of which the high price often charged for rubber bandages is no sufficient guarantee. For varicose veins, for instance, the "Eureka" Bandage may be recommended, for it is porous and easily washable and requires no special skill to adjust. The bandage, in addition, is light, and has additional claims for attention now that the term rubber is used so "elastically" in more senses than one.

### THE INFIRMARY RAZOR.

Through good and evil report, for both attend an innovation, the safety-razor has won its way on the general ground of convenience. Institutional workers, however, are more exacting in their claims, and have found the safety, power of adjustment, and asepsis-securing faculty of the Gillette Safety-Razors a matter of practical experience. The design is simple, there is nothing to get out of order, except the blade, which when its edge is lost is replaced by a new one without a moment's difficulty. It is the principle of the completely new blade, which in the Gillette replaces each dulled one, that has recommended this razor for hospital and institutional use, for the use of a new blade for each operation guarantees complete antisepsis. Nor is the price of new blades such as to make the discarding of old ones in the least expensive. The London address of the Gillette Safety Razor, Ltd., is 40-44 Holborn Viaduct, E.C.

